# Phenotype of Idiopathic Infantile Hypercalcemia Associated with the Heterozygous Pathogenic Variant of *SLC34A1* and *CYP24A1*

**DOI:** 10.3390/children10101701

**Published:** 2023-10-17

**Authors:** Teofana Otilia Bizerea-Moga, Flavia Chisavu, Cristina Ilies, Orsolya Olah, Otilia Marginean, Mihai Gafencu, Gabriela Doros, Ramona Stroescu

**Affiliations:** 1 Department XI of Pediatrics—1st Pediatric Discipline, Center for Research on Growth and Developmental Disorders in Children, ‘Victor Babeș’ University of Medicine and Pharmacy Timișoara, Eftimie Murgu Sq. No. 2, 300041 Timișoara, Romania; bizerea.teofana@umft.ro (T.O.B.-M.); marginean.otilia@umft.ro (O.M.); stroescu@umft.ro (R.S.); 2 1st Pediatric Clinic, ‘Louis Țurcanu’ Children’s Clinical and Emergency Hospital, Iosif Nemoianu 2, 300011 Timișoara, Romania; cristina.ilies@student.umft.ro (C.I.); orsolya.olah@umft.ro (O.O.); 3 4th Pediatric Clinic, ‘Louis Țurcanu’ Children’s Clinical sand Emergency Hospital, Iosif Nemoianu 2, 300011 Timișoara, Romania; mgafencu@umft.ro (M.G.); gdoros@gmail.com (G.D.); 4 Centre for Molecular Research in Nephrology and Vascular Disease, Faculty of Medicine ‘Victor Babes’, 300041 Timișoara, Romania; 5 Department III of Functional Sciences—Pathophysiology Discipline, ‘Victor Babeș’ University of Medicine and Pharmacy Timișoara, Eftimie Murgu Sq. No. 2, 300041 Timișoara, Romania; 6 Department VIII of Neuroscience—Psychology Discipline, ‘Victor Babeș’ University of Medicine and Pharmacy Timișoara, Eftimie Murgu Sq. No. 2, 300041 Timișoara, Romania; 7 Department XI of Pediatrics—3rd Pediatric Discipline, ‘Victor Babeș’ University of Medicine and Pharmacy Timișoara, Eftimie Murgu Sq. No. 2, 300041 Timișoara, Romania

**Keywords:** idiopathic infantile hypercalcemia, nephrocalcinosis, *CYP24A1*, *SLC34A1*

## Abstract

Idiopathic infantile hypercalcemia (IIH) is a rare genetic disease, also called hypersensitivity to vitamin D3. The molecular heterogeneity allows for the differentiation between the two forms; IIH type 1 caused by *CYP24A1* genetic variants and IIH type 2 associated with *SLC34A1* mutations. The affected individuals express a variety of symptoms: hypercalcemia, hypercalciuria, suppressed intact parathormone levels (PTH), nephrocalcinosis, elevated levels of serum 1,25 (OH)2-vitamin D3 or inappropriately normal levels, and kidney phosphate wasting. The present paper describes three cases of IIH with heterozygous mutations in *SLC34A1* and *CYP24A1* genes, respectively. The genetic diagnosis is of paramount importance for proper treatment and the prediction of long-term outcomes.

## 1. Introduction

Idiopathic infantile hypercalcemia (IIH) is a rare condition that can cause severe health effects if not promptly identified. The first description of IHH was in the 1950s in the United Kingdom in infants who had a clinical presentation of severe hypercalcemia [1,2]. The pathophysiology of IIH remained unknown until 2011. In this year, Schlingmann discovered the loss-of-function mutation in the vitamin D catabolizing enzyme 25-OH-vitamin D3-24 hydroxylase (*CYP24A1*) [3]. In the vitamin D activation cascade, the first hydroxylation occurs in the liver by 25-hydroxylase (CYP2R1), with the formation of 25-hydroxyvitamin D3 [4]. In the kidney, a second hydroxylation by 1α-hydroxylase (CYP27B1) creates the active form 1,25-dihydroxyvitamin D3 (1,25-(OH)2-vitamin D3). *CYP24A1* is responsible for the five-step oxidation pathway from 1,25-(OH)2-vitamin D3 to the soluble calcitroic acid by inactivating the 24-hydroxylase (*CYP24A1*) [5,6,7]. Loss-of-function mutations in the *CYP24A1* gene can lead to an increased action due to the accumulation of active 1,25-(OH)2-vitamin D3. The affected individuals express a variety of symptoms: hypercalcemia, hypercalciuria, suppressed intact parathormone levels (PTH), nephrocalcinosis, and increased levels of serum 1,25 (OH)2-vitamin D3 or inappropriately normal levels. In 2016, the genetic heterogeneity of IIH was demonstrated by the recessive mutation discovery in the *SLC34A1* gene, which encodes proximal tubular sodium phosphate co-transported NaPi-II [8]. These patients not only had symptoms of hypercalcemia and suppressed PTH but also exhibited hypophosphatemia due to kidney phosphate wasting and normal vitamin D [3,9].

The molecular heterogeneity allows for the discrimination between the two forms; IIH type 1 caused by *CYP24A1* mutations and IIH type 2 associated with *SLC34A1* genetic variants [10]. The clinical phenotype of IIH is present in the homozygotes, whereas the heterozygotes can display an abnormal phenotype across all ages, with varying symptoms. Common symptoms of IIH in infants include a failure to thrive during the first year of life, vomiting, polyuria, polydipsia, dehydration, constipation, poor feeding, weight loss, hypotonia, and lethargy [11]. The type of inheritance of the disease is still debated since heterozygous individuals present a variety of symptoms from mild forms of hypercalcemia to severe clinical and biochemical hypercalcemia.

The present paper provides a synopsis of the pathological and biological importance of heterozygous carriers of the *CYP24A1* and *SLC34A1* variants in three cases. We analyzed the clinical and biochemical features of these patients that presented early hypercalcemia, nephrocalcinosis, and suppressed levels of the intact parathyroid hormone. The study was approved by the Hospital’s Medical Ethics Committee (89/10 May 2023) in accordance with the World Medical Association’s Ethics Code and informed consent was waived. The data were obtained during hospitalization.

### 1.1. Case Report of IIH Type 1 Phenotype

A 1-year-old male infant was presented with weight loss, poor feeding, and medullary hyperechogenicity on a renal ultrasound. He was the first-born from unrelated parents at 37 gestational weeks, 2600 g, and 52 cm. The infant was breastfed for 6 weeks and continued with standard milk formula. He was given vitamin D (2500 UI/day), lactic calcium, iron, and vitamin B supplements.

**Figure 1:**
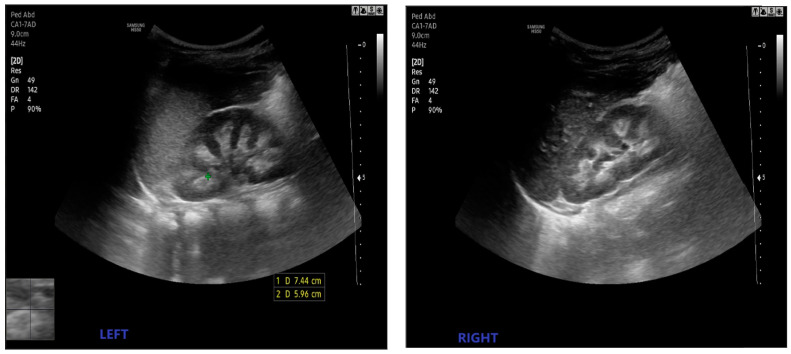
Case 1: ultrasound evaluation of the kidneys, showing the bilateral increased echogenicity of the renal pyramids (**left**, **right**).

Poor feeding, hypotonia, lethargy, irritability, and a failure to thrive were absent. During the first admission, the patient’s weight and height were 8000 g (<P2%) and 80 cm (P50%), respectively. His urine output was 4.68 mL/kg/h. During the physical examination, pallor and macrocrania were noted. The renal ultrasound showed bilateral medullary nephrocalcinosis (Figure 1).

Hypercalcemia was confirmed by the laboratory investigations. The seric levels of the pH-adjusted ionized calcium and total calcium were 1.73 mmol/L (normal range 1.15–1.35 mmol/L) and 3.64 mmol/L (normal range 2.1–2.6 mmol/L). The phosphate level was 1.23 mmol/L (normal range 1.1–2 mmol/L). The blood gas revealed hypokalemic metabolic alkalosis with a pH of 7.51 (normal range 7.35–7.45) and potassium levels of 3.58 mmol/l (normal range 3.6–4.8 mmol/L). The albumin level, which can affect the serum levels of calcium, was normal (46.2 g/L, normal range 3.4–5.4 g/dL). The patient presented high serum creatinine values with normal urea levels. He was diagnosed with acute kidney disease (AKD) stage 2 KDIGO with serum creatinine and urea levels of 37 umol/L and 4.29 mmol/L (normal values 21–36 µmol/L and 1.4–8.3 mmol/L), respectively. Hypercalciuria was identified. The spot urine calcium/creatinine ratio was one (normal value for age < 0.2). A 24-h urine collection could not be performed due to the young age. The intact PTH level was suppressed to < 4.6 pg/mL (normal value 18.5–88.0 pg/mL). The 25-OH-vitamin D was 101.06 ng/mL (normal value 30–100 ng/mL). Normocytic normochromic anemia was identified with a hemoglobin level of 7.7 g/dL (normal value 10.7–14.1 g/dL). An extensive differential diagnosis of anemia was performed, where only higher levels of vitamin B12 were observed; 2250 pg/mL (normal range 211–911 pg/mL). Hyperreninemia was confirmed with a serum renin of 90.36 microUI/mL (normal value 2.8–39.9). Aldosterone was within the normal range. Additionally, the magnesium levels were normal. The patient had normal neurological and motor development. The patient’s mother had a positive history of nephrolithiasis.

Under the suspicion of exogenous vitamin D and vitamin B intoxication, the patient was discharged with the recommendation that all vitamin D, B, and calcium supplements be stopped. At the 3-month follow-up, the patient’s vitamin D, vitamin B12, and total calcium levels dropped to 59.66 ng/mL, 665 pg/mL, and 2.63 mmol/L, respectively. The intact PTH level was still suppressed <4.6 pg/mL. Suspecting infantile hypercalcemia, we conducted the Invitae Nephrolithiasis Panel. The sequence analysis and duplication/deletion testing of the 40 genes are listed in the Genes Analyzed section of Table 1.

One pathogenic variant was seen in *CYP24A1*. *CYP24A1* is associated with autosomal recessive infantile hypercalcemia. A heterozygous pathogenic variant was identified in *CYP24A1* (OMIM 126065) for autosomal recessive IIH type 1 (OMIM 143880) and a heterozygous variant of uncertain significance in KCNJ1 (OMIM 600359) for autosomal recessive Barter syndrome type 2 (OMIM 241200).

The heterozygous pathogenic variant c.1186C>T (p.Arg396Trp) identified in *CYP24A1*, exon 9, occurred in the population databases (rs114368325, gnomAD 0.1%). This missense change was identified in individuals with IIH [3,11,12,13,14,15,16]. In addition, it was segregated with disease in related individuals. Several advanced models of protein biophysical properties and sequences, such as thermodynamic stability, residue mobility, physicochemical variation, spatial, functional and structural information, performed at Invitae suggested that this abnormal variant was expected to disrupt *CYP24A1* protein function. In-vitro studies proved that this missense altered the *CYP24A1* function [3].

The heterozygous of c.553C>T (p.Pro185Ser) of uncertain significance identified in KCNJ1, exon 2 existed in the population databases (rs200154950, gnomAD 0.01%). The variant, also known as p.P166S, has not been reported in the academic field in persons affected by KCNJ1-related conditions. However, the experimental studies proved that this missense change altered the KCNJ1 function [17]. This sequence change replaced two basic neutral amino acids: proline, which is non-polar, with serine, which is polar, at codon 185 of the KCNJ1 protein (p.Pro185Ser). This variant was classified as a variant of uncertain significance because the available evidence was currently insufficient to determine the exact role of it in the disease.

We strongly recommended a genetic analysis of the patient’s relatives and a whole exome sequencing for the patient. However, these tests remain in expectation. The last follow-up, at 3 years old, showed persistent bilateral nephrocalcinosis, a total calcium level of 2.73 mmol/L, normal ionic calcium levels, potassium levels, and blood pH, and a vitamin D level of 42.84 ng/mL. The intact PTH remained suppressed at 8.4 pg/mL, with a slight increase over time. The glomerular filtration rate was normal (162 mL/min/1.73 sm). The anthropometric measurements showed a growth delay (10th percentile for weight and 5th percentile for length). We instructed the patient to maintain a high fluid intake, avoiding exposure to vitamin D (including sun baths if possible), and to sidestep calcium supplements.

### 1.2. Case Report of IIH Type 2 Phenotype

**Figure 2:**
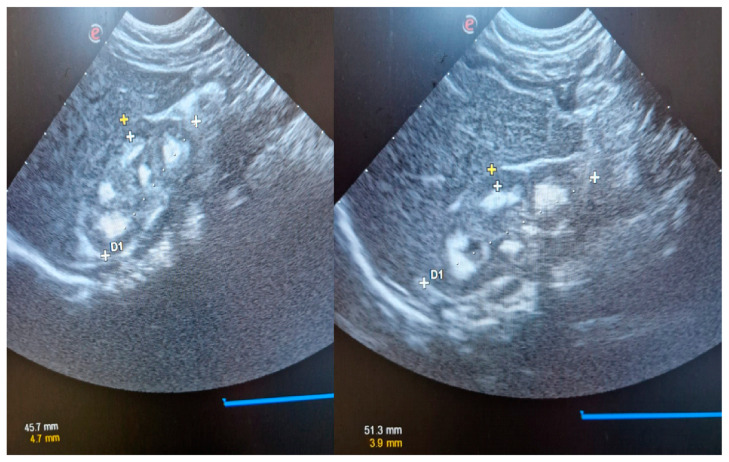
Ultrasound findings in cases two and three, revealing a bilateral hyperechogenicity of the renal pyramids.

Twin girls were presented in our clinic for bilateral medullary hyperechogenicity discovered, incidentally, during the 1-month abdominal echography. The dizygotic twin girls were born prematurely at 33 weeks of gestation from unrelated parents. They received special formula for prematurity and vitamin D supplements (1000 UI/day). At the age of 2 months old, they were admitted into our clinic for investigation. The renal ultrasound findings were consistent with nephrocalcinosis, as shown in Figure 2.

The laboratory investigations revealed a total serum calcium, phosphate, and alkaline phosphatase level of 2.93 mmol/L, 1.01 mmol/L and 1143 U/L, respectively, in twin A and 2.81 mmol/L, 1.03 mmol/L and 1021 U/L, respectively, in twin B. The vitamin D levels were 31.7 ng/mL n twin A and 30.1 ng/mL in twin B, respectively. In both twins, the intact PTH levels were suppressed (<4.6 pg/mL). The blood gas revealed metabolic alkalosis with hypokalemia. In twin A, the serum pH was 7.51, bicarbonate was 28.4 mmol/L, and potassium was 2.91 mmol/L. In twin B, the serum pH was 7.56, bicarbonate the 29.9 mmol/L, and potassium the 2.52 mmol/L. The spot urine calcium/creatinine ratio was 0.31 mg/g in twin A and 0.26 mg/g in twin B. Under the suspicion of IIH, we performed a genetic study of nephrolithiasis and nephrocalcinosis using the Invitae Nephrolithiasis Panel for one twin. The sequence analysis and duplication/deletion testing of the 40 genes are listed in the Genes Analyzed section of Table 1.

The presence of one likely heterozygous pathogenic single nucleotide variant and one heterozygous single nucleotide variant of uncertain clinical significance were identified in the *SLC34A1* gene (OMIM 182309). The c.73C>T p(Arg25Ter) heterozygous variant detected in the *SLC34A1* gene was a nonsense that predicted an amino acid change from arginine to a premature stop codon at position 25. The resulting mRNA would, theoretically, be degraded by the nonsense-mediated decay mechanism, and the loss-of-function variants in this gene were a known pathogenic variant. The c.437C>T p.(Pro146Leu) variant detected in the *SLC34A1* gene was a missense that predicted an amino acid change from proline to leucine at position 146 of the protein. It was described in the clinical databases as a as a pathogenic/likely pathogenic variant. This variant appeared in the dbSNP database (rs200893951) and in the gnomAD population frequency database (0.0061%). The bioinformatic predictor CADD estimated that the change would have a pathogenic effect. The infants were discharged with potassium supplements and a special formula with low calcium and salt contents. Oral Vitamin D supplementation was halted. Genetic counseling was offered to the patient’s parents.

## 2. Discussions

IIH is directly related to an increased synthesis of Vitamin D or a decreased catabolism of active metabolites [3,8]. The recessive mutations of *CYP24A1* responsible for the 25(OH)D3 and 1,25(OH)D3 degradation results in reduced catabolism of the vitamin D active form with all the subsequent consequences of hypervitaminosis. The association between *CYP24A1* with kidney stones and hypercalcemia is characterized by a high variability in the disorder expression as a consequence of environmental and genetic crosstalk. This enzyme deficiency characterizes four to 20% of all calcium kidney stones identified in patients with nephrolithiasis [12].

IIH caused by compound heterozygous or homozygous variants in the *CYP24A1* gene display an autosomal recessive pattern. Our infant presented signs of chronic hypervitaminosis D: macrocrania, hypercalcemia, and suppressed PTH. The associated anemia and metabolic alkalosis with hypokalemia were evaluated and corrected during hospitalization. Surely, the ‘watch and wait’ strategy proved to be a valid approach for the initial management in this case, especially in the presence of high vitamin D levels. The suppressed intact PTH, hypercalciuria, and bilateral nephrocalcinosis imposed the genetic testing. During the sequencing, a pathogenic heterozygous variant was identified in the *CYP24A1* gene. This variant was present in the population databases, including at least one homozygous and/or heterozygous individual. As already mentioned, the patients with calcium kidney stones had an enzyme deficiency of 25-OH-vitamin D3-24 hydroxylase, consistent with their family history (the mother confirmed nephrolithiasis in the past). It has been recognized that heterozygous carrier appeared to have a predisposition for developing nephrolithiasis [3,8].

Infantile hypercalcemia is defined by a failure to thrive, hypercalcemia, hypercalciuria, and nephrocalcinosis in homozygous state. The clinical manifestations depend on the age of diagnosis. Some infants experience a failure to thrive or weight loss, hypotonia, lethargy, dehydration, and vomiting.

However, this was not the case in our patient who presented symptoms at 1 year of age. *CYP24A1*-related hypercalcemia is thought to be a result of increased vitamin D sensitivity, as many infants present symptoms after receiving vitamin D supplementation. The cumulative high doses of vitamin D, calcium supplements, and vitamin B proved to be the trigger for the hypercalcemia symptoms in our case. While some infants with *CYP24A1*-related hypercalcemia quickly have symptoms, some children remain asymptomatic after supplemental vitamin D but demonstrate the biochemical profile of *CYP24A1*-related hypercalcemia [3]. Adults often present renal symptoms, which can include nephrolithiasis, nephrocalcinosis, polyuria, and hypercalcemia, along with other various symptoms. Adults may develop neuropsychiatric disease, gastrointestinal symptoms, hypertension, and pancreatitis [18]. In addition, severe hypercalcemia was described especially during pregnancy or soon after delivery [18].

The heterozygous mutation in KCNJ1, with uncertain significance, probably overlapped the biological parameters severity (metabolic alkalosis with hypopotassemia, hyperreninemia, salt wasting etc.). The KCNJ1 gene is associated with autosomal recessive Bartter syndrome type 2 (BSII). The disease is not caused by all the variants present in a gene. The clinical implication of the variant identified in this gene is questionable. The clinical management decisions based on this result should be performed with caution until this uncertainty is resolved. Our infant associated mild hypokalemia and hyperreninemia without hypertension and metabolic alkalosis, overlapping the clinical signs of hypercalcemia.

All of these findings reinforced the conclusion that the type and the dose of vitamin D administration may unduly modulate adverse effects, thus *CYP24A1*-deficient children were endorsed to restrict the intake of vitamin D. The patient’s phenotype associated the characteristic clinical IIH. Genetic counseling, along with close follow-ups, were required since the patient had chronic kidney disease in the absence of a low glomerular filtration rate. Their biological relatives have increased odds of being a carrier for or being at risk for autosomal recessive infantile hypercalcemia. Testing should be considered if clinically appropriate. The chance of having a child with autosomal recessive infantile hypercalcemia depends on the carrier state of this individual’s partner.

In 2016, a *SLC34A1* gene recessive mutation encoding the sodium phosphate cotransporter NAPi-IIa was identified as responsible for IIH type 2. The primary phosphate wasting induces a disproportionate production of 1,25-(OH)2-vitamin D3 with subsequent hypercalcemia, which reduces promptly after phosphate supplementation. The subsequent statuses of the hyperphosphaturia, hypercalciuria, and hypercalcemia promote renal calcifications due to the formation of calcium phosphate crystals [12,13]. Nephrocalcinosis and/or nephrolithiasis can be identified even in patients with a SLC23A1 heterozygous mutation.

The *SLC34A1* gene (OMIM: 182309) encodes the Solute carrier family 34 member one protein. The pathogenic variants in this gene are associated with Fanconi renotubular syndrome 2 (OMIM: 613388) and infantile hypercalcemia 2 (OMIM: 616963), diseases with a recessive inheritance pattern, and Nephrolithiasis/osteoporosis, hypophosphatemic, 1 (OMIM: 612286), a disease with a dominant inheritance pattern. For those phenotypes associated with an autosomal recessive inheritance pattern, two pathogenic variants in trans configuration (one on each allele) are necessary for diagnostic confirmation.

Since the twins presented idiopathic hypercalciuria, hypercalcemia, and nephrolithiasis, one twin underwent genetic testing for nephrolithiasis and nephrocalcinosis using next-generation sequencing. The heterozygous state of the C.73C>T p.(Arg25Ter) variant detected in the *SLC34A1* gene of the twin was described in the clinical database as a pathogenic/likely pathogenic variant with a frequency of 0.0061% [19,20,21]. Although the c.437C>T p.(Pro146Leu) variant of uncertain clinical significance detected in the *SLC34A1* gene was considered benign, this variant was detected in heterozygosity in two patients with nephrocalcinosis and idiopathic infantile hypercalcemia [22,23]. It was recommended to determine the disposition of the changes (cis/trans) in the SCL34A1 gene in the first degree relatives to determine a possible correlation with the twins’ phenotype. Unfortunately, our country does not provide special milk formulas or genetic testing for caregivers. Genetic counseling was offered to the patient’s parents. The presence of one heterozygous likely pathogenic single nucleotide variant and one heterozygous single nucleotide variant of uncertain clinical significance did not allow for confirming or ruling out the suspected clinical diagnosis from the genetic point of view. This was due to the insufficient evidence of causality, alterations not detectable by the technique that was used, or the presence of variants in the other genes that were not analyzed.

Ultrasound is of paramount importance for diagnosing nephrocalcinosis, as it allows for a non-invasive visualization of calcium deposits in the kidneys, enabling early detection and timely intervention to prevent further kidney damage. However, multiparametric ultrasound that combines various ultrasound modalities, like advanced doppler techniques, contrast enhanced ultrasound, and stiffness measurements using elastographic methods, should be considered to enhance the accuracy and diagnostic capabilities when identifying and characterizing nephrocalcinosis [24].

The progression and long-term consequences of IIH remain relatively unclear. Although, the clinical symptoms have been shown to resolve spontaneously as serum calcium levels return to normal. A study conducted on 18 IIH patients demonstrated a less favorable renal prognosis compared to the general population. Among these individuals, a high occurrence of chronic kidney disease (CKD) was observed (77% of cases were classified as CKD II), and two patients were diagnosed with end-stage renal disease despite efforts to avoid vitamin D or calcium supplementation and sun exposure [23,25,26].

## 3. Conclusions

The implementation of genetic testing for *SLC34A1* and *CYP24A1* in early-onset nephrocalcinosis pediatric patients is a valuable tool for proper short and long-term management of the kidney–parathyroid–bone axis. Long-term follow-up is mandatory in this study, as these specific patients have early-onset chronic kidney disease with a preserved glomerular filtration rate. The patient’s phenotype was associated with the clinical signs of IIH even in the heterozygous state.

## Figures and Tables

**Table 1:** Genes Analyzed section.

Gene	NM	Mean Coverage	10× (%)	Gene	NM	Mean Coverage	10× (%)
*ADCY10*	NM_018417	130.59	100	*HPRT1*	NM_000194	324.17	100
*AGXT*	NM_000030	86.23	100	*KCNJ1*	NM_153766	131.96	100
*APRT*	NM_000485	71.86	100	*OCRL*	NM_000276	242.82	100
*ATP6V0A4*	NM_020632	125.53	100	*SLC12A1*	NM_000338	121.32	100
*ATP6V1B1*	NM_001692	97.72	100	*SLC22A12*	NM_144585	90.27	100
*CA2*	NM_000067	104.32	100	*ALC26A1*	NM_022042	78.55	100
*CASR*	NM_000388	90.22	100	*SLC2A9*	NM_020041	96.87	100
*CLCN5*	NM_001127898	203	100	*SLC34A1*	NM_003052	79.45	100
*CLDN16*	NM_006580	112.14	100	*ALC34A3*	NM_001177316	64.24	99.85
*CLDN19*	NM_148960	91.37	100	*SLC3A1*	NM_000341	139.9	100
*CYP24A1*	NM_000782	111.66	100	*SLC4A1*	NM_000342	85.81	100
*FAM20A*	NM_017565	105.43	100	*ALC7A9*	NM_014270	108.73	100
*GRHPR*	NM_012203	99.34	100	*SLC9A3R1*	NM_004252	76.37	100
*HNF4A*	NM_175914	101.09	100	*VDR*	NM_000376	84.2	100
*HOGA1*	NM_138413	101	100	*XHD*	NM_000379	112.17	100

## Data Availability

The data collected for this study will be available upon request from the corresponding authors. The data that will be available is represented by de-identified participant data. The inform consent form will be available upon request. The data will be available with the publication. The data will be available by request at the e-mail address farkasflavia8@gmail.com. The data will be shared after direct request and after approval of the proposal by all the authors.
